# ALS-specific cognitive and behavior changes associated with advancing disease stage in ALS

**DOI:** 10.1212/WNL.0000000000006317

**Published:** 2018-10-09

**Authors:** Christopher Crockford, Judith Newton, Katie Lonergan, Theresa Chiwera, Tom Booth, Siddharthan Chandran, Shuna Colville, Mark Heverin, Iain Mays, Suvankar Pal, Niall Pender, Marta Pinto-Grau, Ratko Radakovic, Christopher E. Shaw, Laura Stephenson, Robert Swingler, Alice Vajda, Ammar Al-Chalabi, Orla Hardiman, Sharon Abrahams

**Affiliations:** From Human Cognitive Neuroscience (C.C., J.N., T.B., R.R., S.A.), Psychology, Philosophy, Psychology and Language Sciences, Euan MacDonald Centre for Motor Neurone Disease Research (C.C., S.A.), and Alzheimer Scotland Dementia Research Centre (R.R.), University of Edinburgh; Anne Rowling Regenerative Neurology Clinic (J.N., S.C., S.P., R.R., L.S., R.S., S.A.), Royal Infirmary of Edinburgh, UK; Academic Unit of Neurology (K.L., M.H., I.M., M.P.-G., A.V., O.H.), Trinity College Dublin; Departments of Psychology (K.L., I.M., N.P., M.P.-G.) and Neurology (O.H.), Beaumont Hospital, Dublin, Ireland; and Maurice Wohl Clinical Neuroscience Institute (T.C., C.E.S., A.A.-C.), Department of Basic and Clinical Neuroscience, King's College London, UK.

## Abstract

**Objective:**

To elucidate the relationship between disease stage in amyotrophic lateral sclerosis (ALS), as measured with the King's Clinical Staging System, and cognitive and behavioral change, measured with the Edinburgh Cognitive and Behavioural ALS Screen (ECAS).

**Methods:**

A large multicenter observational cohort of 161 cross-sectional patients with ALS and 80 healthy matched controls were recruited across 3 research sites (Dublin, Edinburgh, and London). Participants were administered the ECAS and categorized into independent groups based on their King's clinical disease stage at time of testing.

**Results:**

Significant differences were observed between patients and controls on all subtests of the ECAS except for visuospatial functioning. A significant cross-sectional effect was observed across disease stages for ALS-specific functions (executive, language, letter fluency) and ECAS total score but not for ALS-nonspecific functions (memory, visuospatial). Rates of ALS-specific impairment and behavioral change were also related to disease stage. The relationship between cognitive function and disease stage may be due to letter fluency impairment, whereas higher rates of all behavioral domains were seen in later King's stage. The presence of bulbar signs, but not site of onset, was significantly related to ALS-specific, ECAS total, and behavioral scores.

**Conclusion:**

ALS-specific cognitive deficits and behavioral impairment are more frequent with more severe disease stage. By end-stage disease, only a small percentage of patients are free of neuropsychological impairment. The presence of bulbar symptoms exaggerates the differences observed between disease stages. These findings suggest that cognitive and behavioral change should be incorporated into ALS diagnostic criteria and should be included in future staging systems.

Amyotrophic lateral sclerosis (ALS) is marked by progressive degeneration of motor neurons, with death usually occurring 2 to 3 years from onset.^1^ Approximately 35% of patients with ALS experience cognitive or behavioral impairment, with an additional 15% having frontotemporal dementia.^2,3^

Executive dysfunction is commonly reported in ALS, in addition to impairment in language and social cognition,^3–7^ whereas apathy is the most frequently reported behavioral feature.^8,9^ Longitudinal studies of cognition in ALS have been confounded by small numbers, the use of clinic-based populations, and attrition.^10–12^ However, existing data^13^ indicate that cognitive change may relate to indirect measures of disease progression (e.g., total score on the ALS Functional Rating Scale–Revised [ALSFRS-R]), suggesting that this third domain should be included in diagnostic criteria and staging systems such as the King's Clinical Staging System.^14^

The objective of this study was to examine the clinical presentation of cognitive and behavioral symptoms across different disease stages of ALS as defined by the King's Clinical Staging System. Specifically, the aim was to examine whether cognition and behavior are related to advancing disease stage in a clinically representative sample of patients with ALS, which domains of cognition and behavior are particularly related to disease stage, and which, if any, clinical variables relate to cognition and behavior in ALS.

## Methods

### Standard protocol approvals, registrations, and patient consents

This study is a multicenter cross-sectional observational study. All participants provided informed written consent, and this research was approved by the South-East Scotland Research Ethics Committee and the Medical Research Ethics Committee of Beaumont Hospital, Dublin.

### Participants

One hundred sixty-one patients meeting revised El Escorial diagnostic criteria for possible, probable, or definite ALS^15^ were included. Patients were prospectively recruited across 3 research centers in Edinburgh, Dublin, and London between July 2014 and July 2016. Of the patients recruited, 88.8% were incident cases (n = 143) being assessed within 12 months of diagnosis. Recruitment was population based in Dublin and through ALS clinics in Edinburgh and London. Exclusion criteria included a history of dyslexia, marked premorbid reading, or writing difficulties or a learning disability; nonfluent premorbid English reading and writing abilities; history of other neurologic conditions that could affect cognition such as major hemispheric stroke, traumatic brain injury, and severe active epilepsy; alcohol and drug dependencies; and severe physical disability or weakness at the time of assessment prohibiting participation. Of the 161 participants with ALS, 149 primary caregivers consented to provide behavioral data. Eighty demographically matched healthy adults were additionally recruited as a control group. Healthy controls met the same inclusion criteria as the patient group and were not a blood relative of a person with ALS. The control group was recruited through research volunteer panels held by the University of Edinburgh and Trinity College Dublin, non–blood relatives of patients with ALS, and local community noticeboards.

### Procedure and materials

Clinic- and home-based semistructured interviews were conducted to collect demographic and clinical data. Socioeconomic status was measured with the National Statistics Socio-Economic Classification Self-Coded Scale (Standard Occupational Classification, 2010) modified to include the category of long-term unemployed. Functional status was assessed with the ALSFRS-R.^16^ Mood was measured with a modified version of the Hospital Anxiety and Depression Scale, which excludes items confounded by motor disability.^17,18^

Clinical staging was measured with the King's Clinical Staging System.^14,19^ Each stage of the disease is based on regions of involvement; regions are bulbar, upper limbs, lower limbs, and respiratory or nutritional domains. Stage 1 is defined as the involvement of 1 bodily region (e.g., an upper limb); stage 2 is defined as the involvement of 2 bodily regions (e.g., upper limb and lower limb); stage 3 is defined as involvement of 3 bodily regions (i.e., upper limb, lower limb, and bulbar); and stage 4 is defined as respiratory or nutritional insufficiency requiring intervention. Regional involvement was determined by the presence of functional signs (e.g., changes in speech) or clinical examination (e.g., fasciculations, wasting of first dorsal interosseous). Respiratory and nutritional insufficiency was determined as per the National Institute for Health and Care Excellence guidelines for motor neuron disease assessment and management,^20^ including arterialized capillary blood gas tensions, nocturnal arterial oxygen saturation, forced vital capacity, or sniff nasal inspiratory pressure. The King's system has demonstrated good prognostic utility, providing a linear and standardized metric of disease progression.^14,19,21^

Neuropsychological status was measured with the Edinburgh Cognitive and Behavioural ALS Screen (ECAS).^22^ The ECAS is independent of motor disability and consists of 15 subtests across 5 cognitive domains: language functions, executive functions, and letter fluency combine to generate a composite ALS-specific score, while memory and visuospatial functioning combine to form an ALS-nonspecific score. The ECAS also consists of a caregiver behavioral interview based on the Rascovsky criteria for behavioral variant frontotemporal dementia.^23^ The behavior interview is a structured clinical interview conducted in private with patients' caregivers. The interview measures 5 domains of behavior: behavioral disinhibition; loss of sympathy/empathy; apathy or inertia; perseverative, stereotyped, or compulsive/ritualistic behaviors; or hyperorality and dietary changes. The behavior interview additionally includes 3 questions measuring the presence of psychotic features. Behavioral data were gathered at the Dublin site, and the presence/absence of behavior features was supported by the Beaumont Behaviour Inventory.^24^ The ECAS was selected as the primary outcome measure to reduce the burden of participation by its brevity and independence of motor speed, thereby reducing bias in participation.

### Statistical analyses

Demographic, clinical, and neuropsychological data for the patient and control groups were compared with a χ^2^ test for categorical data (or Fisher exact test when expected cell frequencies fell below 5) or Welch *t* tests and one-way analyses of variance for continuous data. Effect sizes for group comparisons were calculated from η^2^, the Cramer *V*, and *r* for Mann-Whitney *U* tests. The relationships among ECAS subdomains were explored with tetrachoric correlation analysis. To examine whether cognition or behavior is related to disease stage, patients with ALS were divided into independent groups based on their King's clinical disease stage at time of testing. Jonckheere-Terpstra tests were used on raw ECAS scores specifying a decreasing trend for cognition and an increasing trend for behavior with *p* values approximated under the central limit theorem for 10,000 permutations. The ALS-specific, ALS-nonspecific, and ECAS total scores were the primary cognitive outcome measures because of their high sensitivity to cognitive impairment against a full neuropsychological battery.^25,26^ The number of reported behavior domains (maximum 5) of the ECAS behavior interview was the primary behavioral outcome measure. When significant relationships were observed, the respective ALS-specific (language, executive, and fluency), ALS-nonspecific (memory and visuospatial), and behavior (apathy, disinhibition, loss of sympathy/empathy, perseverative, and eating behaviors) subdomains were analyzed to explore the nature of this relationship.

Cognitive impairment was determined from local validated abnormality cutoff scores from UK and Irish populations.^25,26^ Behavioral impairment was defined as the presence of ≥2 behavioral features or the presence of apathy, as described by the recent consensus guidelines for diagnosing frontotemporal spectrum disorder.^27^ Rates of impairment between disease stages were analyzed with the Cochran-Armitage test, which evaluates the significance of an increasing binomial proportions trend across an ordinal grouping variable.

The relationship between neuropsychological performance and clinical variables was also explored with 1-way analysis of variance, Wilcoxon Mann-Whitney, and Spearman correlation tests. For all analyses, when data violated statistical assumptions, log or power transformation was applied. When transformation failed to correct violations, nonparametric alternatives were used. Multiple comparisons were corrected for with the Holm-Bonferroni method. Missing values were excluded pairwise unless otherwise stated. Analyses were conducted with R 3.3.2 (R Foundation for Statistical Computing, Vienna, Austria) with α set to 0.05.

### Data availability

Anonymized data presented in this article will be made available at the request of a qualified investigator. Requests should be made to Sharon Abrahams (s.abrahams@ed.ac.uk). Supplemental data are available at http://dx.doi.org/10.7488/ds/2422.

## Results

Demographic data on patients with ALS and controls are presented in table 1. No significant differences were observed between the patient and control groups for background variables or levels of depression and anxiety. Sixty-four percent (n = 103) of patients had classic ALS with symptom onset in the upper or lower limbs; 26% (n = 41) had bulbar onset; 9% (n = 15) had mixed onset; and 1% (n = 2) had respiratory onset.

**Table 1:**
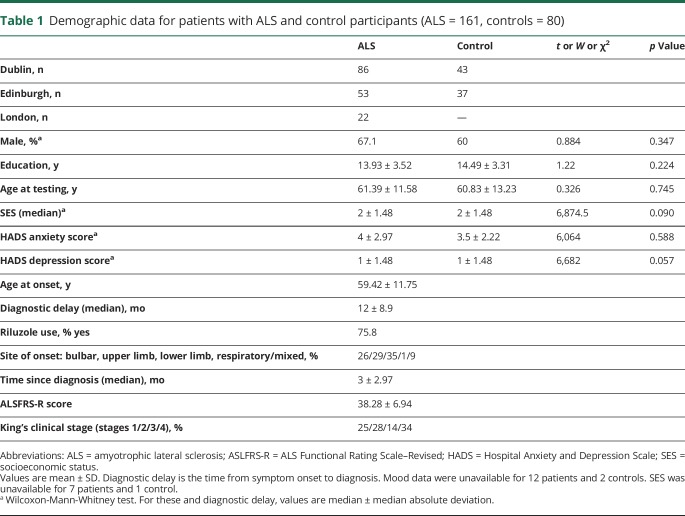
Demographic data for patients with ALS and control participants (ALS = 161, controls = 80)

The cognitive performance of patients with ALS was compared to that of the control group for each domain of the ECAS. Significant differences were observed for language, executive functions, letter fluency, and memory, while no significant difference was observed for visuospatial functioning. The composite ALS-specific, ALS-nonspecific, and ECAS total scores all demonstrated significant between-group differences (data available from Edinburgh DataShare, table e-1, http://dx.doi.org/10.7488/ds/2422); 28.5% of patients were found to have cognitive impairment on the ECAS total, 27% on ALS-specific, and 19.4% on ALS-nonspecific scores. Letter fluency impairment was most commonly observed (30.4%), followed by executive (22.5%) and language (21.3%) dysfunction. Memory (16.8%) and visuospatial (9.4%) impairment was less commonly found.

Of the 149 patients for whom behavioral data were available, 45% had no behavioral features, 21.5% had 1 feature, 14.1% had 2 features, and 19.5% had ≥3 features. Behavioral impairment as described by the revised consensus guidelines^27^ was found in 39.6% of patients. Apathy was the most commonly reported behavioral feature (30.9%), followed by a loss of sympathy/empathy (27.5%), changes in eating behaviors (24.8%), perseveration (24.8%), and disinhibition (15.4%).

Impairment in cognitive domains was most strongly associated with other cognitive domains rather than behavioral features and vice versa (data available from Edinburgh DataShare, table e-2 and figure e-1, http://dx.doi.org/10.7488/ds/2422). Language, fluency, executive, and memory impairment co-occurred (*r*_tet_ = 0.27–0.49). Similarly, the co-occurrence of behavioral features was strong, ranging from 0.37 to 0.79. The relationship between cognition and behavior was weaker, with a few exceptions. Relationships were observed between sympathy/empathy and executive dysfunction (*r*_tet_ = 0.37), disinhibition and fluency impairment (*r*_tet_ = 0.38), and visuospatial impairment and perseveration (*r*_tet_ = 0.44) and hyperorality (*r*_tet_ = 0.35).

### Cognition, behavior, and King's clinical disease staging

Patients were divided into their respective King's clinical stage at time of testing. Demographic and clinical variables are described for each disease stage group in table 2. No significant differences were observed between the 4 patient groups for most variables. As expected, ALSFRS-R scores significantly differed between disease stages (*F*_3,146_ = 25.97, *p* < 0.0001, η^2^ = 0.348). A significant dependency was observed between site of onset and disease stage [χ^2^(6) = 17.38, *p* = 0.008, *V* = 0.247], driven by a higher proportion of patients with bulbar onset compared with upper limb onset in stages 1 and 4 (standardized residuals 1.44 and 1.42) and the inverse for stages 2 and 3 (residuals −1.80 and −1.48, respectively). Differing levels of depressive symptoms as measured by the Hospital Anxiety and Depression Scale were observed across disease stages [H(3) = 18.18, *p* < 0.001]. Post hoc analysis showed that stage 1 significantly differed from stage 2 (*p* = 0.043, *r* = 0.262) and stage 4 (*p* < 0.001, *r* = 0.430).

**Table 2:**
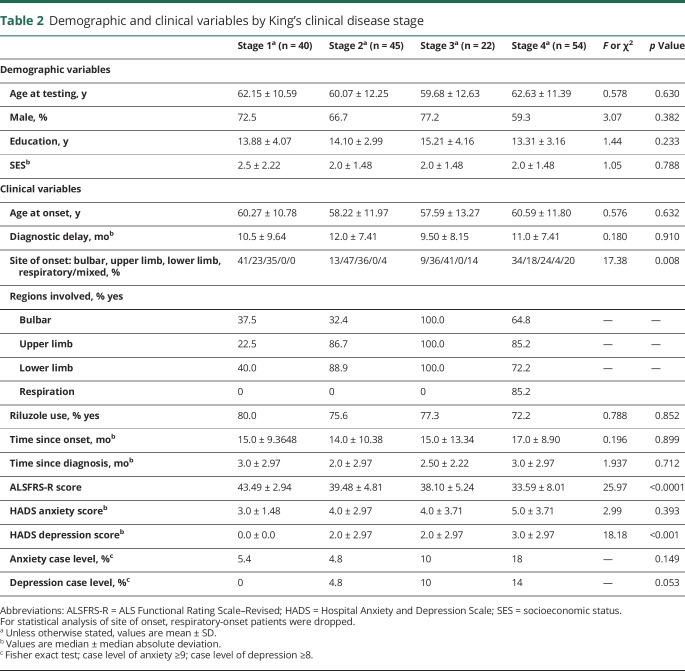
Demographic and clinical variables by King's clinical disease stage

**Figure 1:**
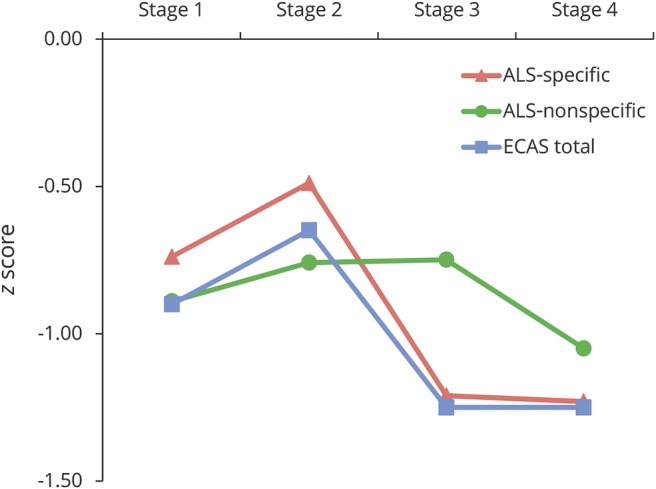
Cognitive performance across King’s clinical disease stages Patient performance is scaled to a standardized score (*z* score) on the basis of the mean and SD of local UK and Irish control groups. ALS = amyotrophic lateral sclerosis; ECAS = Edinburgh Cognitive and Behavioural ALS Screen.

**Figure 2:**
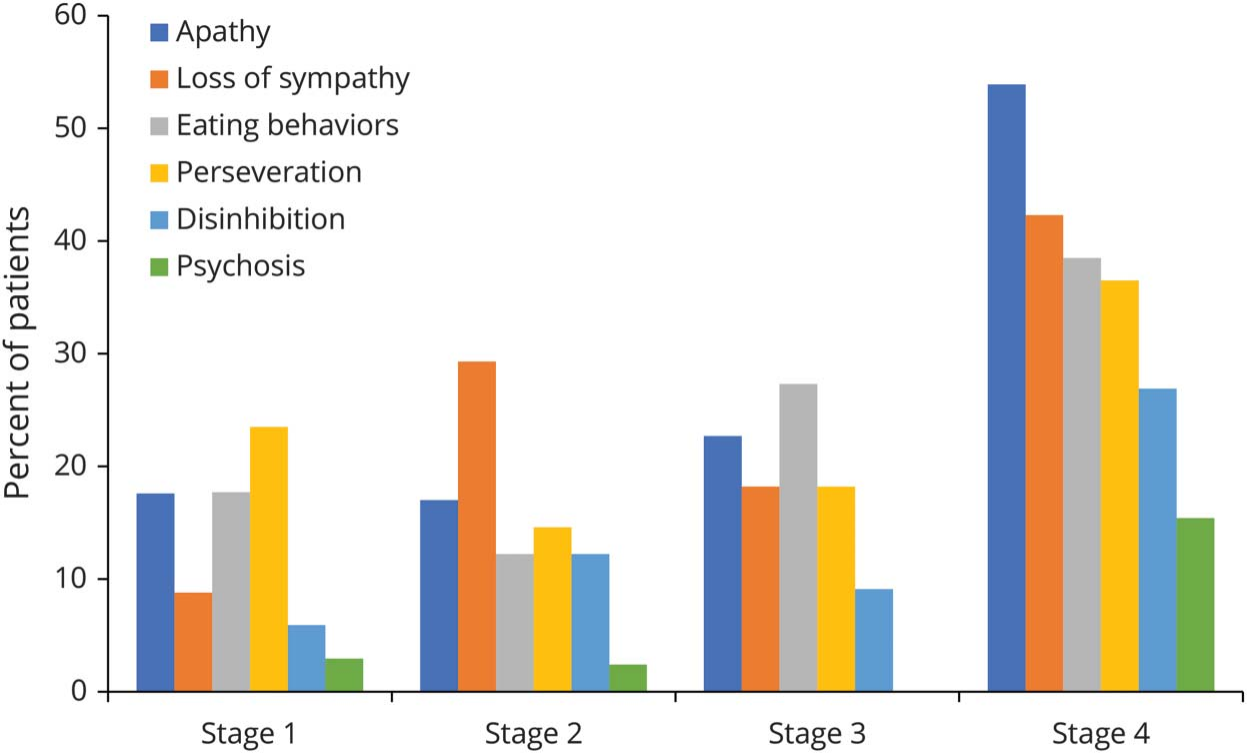
Frequency of behavioral impairment across King's clinical disease stages

Cognitive performance (represented as a *z* score calculated against local normative data) for patients within each disease stage is presented in figure 1, with raw scores presented in table 3. To explore whether cognitive and behavioral performance differs between disease stages, Jonckheere-Terpstra tests were used on ECAS raw scores. A significant effect, corrected for multiple comparisons, was observed for ALS-specific score (T_JT_ = 3,804.5, *p* = 0.022), ECAS total score (T_JT_ = 3,845.5, *p* = 0.026), and number of behavioral features (T_JT_ = 5,295.5, *p* < 0.001), demonstrating lower cognitive ability and a higher number of behavior features across advancing disease stages. No significant effect was observed for ALS-nonspecific functions. To examine which domains of ALS-specific functions were driving this result, analysis of the ALS-specific and behavioral subdomains was conducted. Executive function (T_JT_ = 4,061, *p* = 0.035) and letter fluency (T_JT_ = 3,721.5, *p* = 0.001) scores significantly related to more advanced disease stages; however, after correction for multiple comparisons, only letter fluency remained significant (*p* = 0.002). Analysis of the behavioral domains showed that apathy (*z* = 4.00, *p* < 0.001), disinhibition (*z* = 2.65, *p* = 0.012), loss of sympathy or empathy (*z* = 3.06, *p* = 0.005), perseveration (*z* = 1.68, *p* = 0.036), and eating behaviors (*z* = 2.76, *p* = 0.012) were significantly related to disease stages after correction for multiple comparisons (figure 2). The presence of psychotic features was also more common in later disease stages (*z* = 2.45, *p* = 0.014). Thus, cognitive functions specific to ALS (particularly letter fluency), behavior (apathy, disinhibition, loss of sympathy/empathy, perseveration, and disinhibition), and psychosis are significantly associated with disease stage, with later stages relating to more severe neuropsychological symptoms. These findings are consistent when the sample is restricted to incident cases only.

**Table 3:**
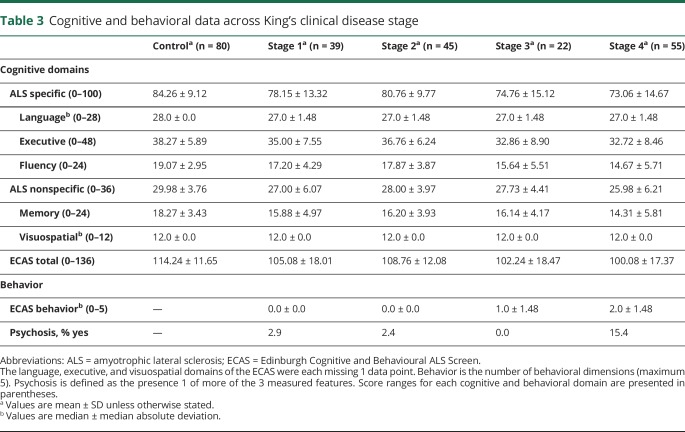
Cognitive and behavioral data across King's clinical disease stage

While stage 4 is a marker of end-stage disease, it may be considered a prognostic indicator rather than indicating more severe spread of pathology. Therefore, data were reanalyzed with the exclusion of stage 4 patients without bulbar, upper limb, and lower limb involvement (n = 130), with the majority of results unchanged. The significant effect for ECAS total (T_JT_ = 2,514, *p* = 0.039), ALS-specific functions (T_JT_ = 2,477.5, *p* = 0.017), and behavior (T_JT_ = 3,438, *p* < 0.001) persisted, with behavior surviving correction for multiple comparisons (*p* < 0.001). Examination of the ALS-specific domains reveals that letter fluency is significant after correction (T_JT_ = 2,483, *p* = 0.020). Cochran-Armitage tests of behavior domains reveal that apathy (*z* = 2.85, *p* = 0.009), disinhibition (*z* = 3.73, *p* < 0.001), loss of sympathy or empathy (*z* = 3.15, *p* = 0.004), eating behaviors (*z* = 2.51, *p* = 0.018), and psychosis (*z* = 2.07, *p* = 0.039) remained significant. Reanalysis of data with stage 4 removed entirely reveals no significant effect for disease stage.

### Rates of neuropsychological impairment and King's clinical disease stage

**Figure 3:**
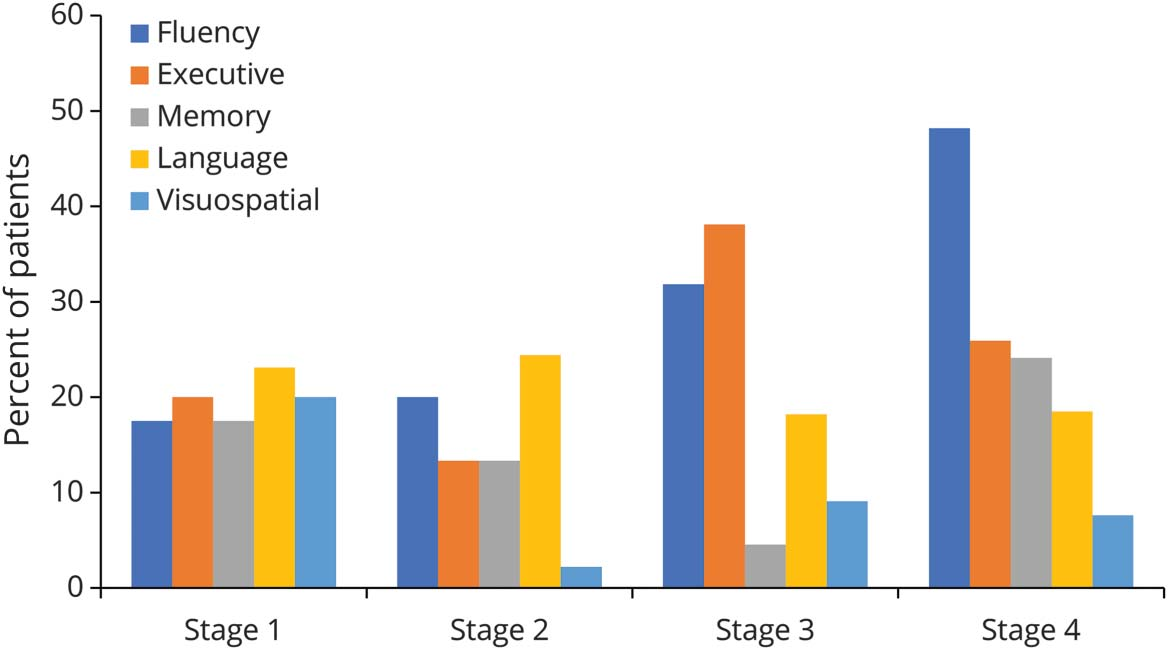
Frequencies of impairment across King's clinical disease stage for Edinburgh Cognitive and Behavioural ALS Screen cognitive domains

Consistent with the analyses of the raw scores, a significant effect for higher rates of impairment was observed across disease stages for ALS-specific functions after correction for multiple comparisons (table 4). Of the ALS-specific subdomains, a significant relationship was observed (*z* = 3.54, *p* < 0.001) for letter fluency impairment (figure 3). While rates of impairment for ALS-nonspecific functions differed between stages 3 and 4, this did not reach statistical significance. Rates of behavioral impairment were significantly higher in more advanced disease stages.

**Table 4:**
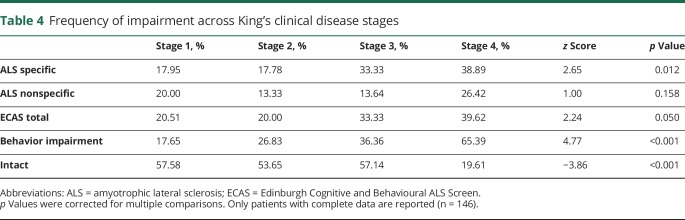
Frequency of impairment across King's clinical disease stages

Patients were classified as neuropsychologically intact if there was no evidence of behavioral impairment and no evidence of cognitive impairment (ALS-specific, ALS-nonspecific, ECAS total scores). Patients for whom behavioral data were unavailable were not included in this classification. A significant effect was found for lower rates of neuropsychologically intact patients such that by stage 4 only 19.6% of patients were free of impairment. The effect of disease stage on rates of impairment did not change when data were restricted to incident cases. No change in results was observed when stage 4 patients without concurrent involvement of bulbar, upper limb, and lower limb regions were removed. Results did not survive the removal of stage 4 patients.

Patients with and without neuropsychological impairment were compared on demographic and clinical variables (described in table 1) to determine which, if any, distinguish the groups. For demographic information, only education significantly differed between patients with and those without neuropsychological impairment [*t*(123.93) = −2.44, *p* = 0.016]. However, after correction for multiple comparisons, this was no longer significant (*p* = 0.065). Regarding clinical features, anxiety (*W* = 2,941, *p* = 0.013), depression (*W* = 3,359.5, *p* < 0.001), and ALSFRS-R score [*t*(133.92) = −3.55, *p* = 0.004] significantly differed between groups. Thus, patients with neuropsychological impairment have higher levels of depression and anxiety, lower ALSFRS-R scores, and potentially fewer years of education.

### Cognition, behavior, and clinical variables

Clinical variables were analyzed against ALS-specific, ALS-nonspecific, and ECAS total scores and the number of behavioral features present (data available from Edinburgh DataShare, tables e-2 and e-3, http://dx.doi.org/10.7488/ds/2422). A significant relationship was observed between the presence of bulbar involvement (but not site of onset) and ALS-specific functions (*W* = 3,896.5, *p* = 0.033) and behavior (*W* = 2061, *p* = 0.019). Patients were subdivided within each stage on the basis of the presence or absence of bulbar involvement (data available from Edinburgh DataShare, tables e-4 and e-5, http://dx.doi.org/10.7488/ds/2422). Subsequent Jonckheere-Terpstra tests revealed that patients with evidence of bulbar involvement demonstrated significantly worse ALS-specific (T_JT_ = 887, *p* = 0.021), ALS-nonspecific (T_JT_ = 956, *p* = 0.028), ECAS total (T_JT_ = 875, *p* = 0.021) scores and behavioral features (T_JT_ = 1,583, *p* < 0.001) after correction for multiple comparisons. Conversely, patients without bulbar signs demonstrated no significant relationship in cognitive or behavioral features.

Depression (*r*_s_ = 0.3560, *p* < 0.001) and ALSFRS-R score (*r*_s_ = −0.258, *p* = 0.009) were related to behavior. To explore which behavioral domain was related to depression, depression scores of patients with and without each behavioral feature were compared. Significant differences, after correction, were observed for patients with and without apathy (*W* = 1,043, *p* < 0.001), disinhibition (*W* = 773.5, *p* = 0.018), and loss of sympathy/empathy (*W* = 1,036, *p* < 0.001). No significant relationship was observed between cognition or behavior and site of onset, diagnostic delay, riluzole use, weight, upper limb involvement, lower limb involvement, or levels of anxiety.

## Discussion

The aim of the present study was to determine the relationship between cognitive and behavioral symptoms as measured with the ECAS and the King's Clinical Staging System. In particular, the present study aimed to evaluate whether cognition and behavior are related to advancing disease stage in a clinically representative sample of patients with ALS, which domains of cognition and behavior are particularly related to disease stage, and which, if any, clinical variables relate to cognition and behavior in ALS.

Our findings demonstrated that cognitive domains that are typically affected in ALS (ALS-specific), the ECAS total performance, and the number of reported behavioral features are significantly related to King's clinical disease stages. Conversely, no such association was observed for cognitive functions not typically affected by ALS (i.e., memory and visuospatial functioning). Behavioral impairment as defined by the newly updated Strong et al.^27^ criteria also was related to disease stage, with all 5 ECAS behavioral domains demonstrating increasing impairment in more advanced stages. These findings demonstrate that ALS-specific cognitive functioning and behavior are significantly and negatively related to advancing disease stage. This relationship is driven most strongly by letter fluency performance, with executive dysfunction possibly also playing a role and global behavioral changes across all types of behavior that characterize behavioral variant frontotemporal dementia.^23^

Structural and functional neuroimaging has shown that changes in ALS include extramotor areas that are involved in higher-order cognitive processing and behavioral control (see reference 28 for overview). Executive functioning (including social cognition), fluency, and language have been associated with dysfunction of frontal and temporal regions of brain. For example, executive functioning and social cognition in ALS have been related to prefrontal dysfunction in ALS,^29,30^ with white matter tract connectivity also implicated.^31–34^ Letter fluency is a sensitive marker of cognitive impairment in ALS and has similarly been linked to prefrontal dysfunction.^5,29^ Neuropsychological studies have shown that letter fluency impairment may represent a difficulty in cognitive initiation,^4,35^ which in turn is related to the high frequency of apathy in ALS.^36^ Similar to executive functioning and letter fluency, apathy has been associated with reduced fractional anisotropy in the right anterior cingulate cortex^37^ and the dorsolateral and orbitomedial prefrontal cortex.^38^ Pathologic TAR DNA-binding protein 43 inclusions have been suggested to spread predictably in ALS,^39^ beginning in the primary motor cortex, spinal cord, and cranial nerves and spreading to the reticular formation of the brainstem, prefrontal cortex, and finally hippocampus. Executive and fluency dysfunction is commonly reported in ALS, possibly because of early pathologic involvement of the prefrontal cortex. However, memory dysfunction is less commonly reported, perhaps resulting from the exclusion of patients with end-stage ALS from research studies (i.e., those with respiratory insufficiency). Indeed, memory impairment may be a feature of end-stage ALS, but it currently is underrecognized. The strength of the relationship between behavior and disease stage may suggest that behavior is more susceptible to pathologic disease spread than cognition. Higher rates of cognitive and behavioral dysfunction across disease stage therefore implicate progressive involvement of frontotemporal regions. However, given that respiratory dysfunction is one of the defining features of disease stage 4, the late-stage involvement of ALS-nonspecific (e.g., memory) functions may be associated with declining respiratory function, which could be ameliorated by appropriately prescribed ventilatory support.

Previous cross-sectional and longitudinal research on cognition in ALS has been inconsistent as to whether cognition declines. Clinic-based studies have failed to reliably observe a relationship between cognition and disease progression.^10–12^ A large population-based longitudinal study from our group has previously shown a relationship between the ALSFRS-R and cognition.^13^ This inconsistency is most likely a function of sample sizes, high attrition rates, clinic- vs population-based sampling, incident vs prevalence sampling, and the variability in metrics used to approximate disease progression (i.e., time or the ALSFRS-R).

Because ALS is a heterogeneous condition with different disease trajectories, a system that defines progression based on clinical decline rather than as a function of time since first presentation is of greater utility when disease progression is analyzed. The King's Clinical Staging System is designed to overcome variability in disease trajectory over time. Our findings of a relationship between ALS-specific cognitive and behavioral change and King's clinical disease stage provide additional evidence of spread of degenerative processes in the prefrontal cortices.

These findings have important clinical implications, with neuropsychological impairment previously associated with reduced survival,^40,41^ quality of life,^42,43^ caregiver burden,^44,45^ and the ability to manage and engage in life-prolonging interventions.^46,47^ It is therefore possible that quality of life and caregiver burden may also relate to disease stage. Clinically, it may be necessary to consider intervention programs for caregivers to alleviate the impact of neuropsychological impairment, particularly early in the disease course. Furthermore, clinicians should be cognizant of current neuropsychological status when prescribing life-prolonging interventions to patients and implement support structures for those with a neuropsychological impairment, e.g., by providing instructions in simple written or pictorial format to reduce cognitive burden. The relationship between disease stage and behavior is of particular importance given the strength of this relationship relative to cognition and its negative impact on patients and caregivers. Behavior change is less commonly reported in the literature compared to cognition and is often reported as a unidimensional construct. The profile and impact of behavioral change merit further and more detailed investigation in the future. Thus, monitoring of both cognitive and behavioral symptoms across the disease course is vital to providing appropriate and timely care and support to patients with ALS and their families.

Consequently, the recently updated UK National Institute for Health and Care Excellence^20^ guidelines on motor neuron disease assessment and management have incorporated cognitive and behavioral assessment as integral factors in patient care. Furthermore, the majority of patients with ALS and caregivers have expressed their desire to be informed about the risk of neuropsychological impairment from their physician.^48^ We have found that 80% of patients in King's stage 4 experience cognitive or behavioral impairment. The relatively low frequency of cognitively intact patients argues in favor of incorporating cognitive and behavioral screening as a standard measure in ALS assessment.

We found no significant relationship between cognition, behavior and diagnostic delay, riluzole use, weight at testing, upper limb involvement, or lower limb involvement. However, the present findings suggest that bulbar involvement (but not site of onset) significantly relates to cognitive and behavioral performance. The relationship between the presence of bulbar symptoms and cognition has been suggested previously.^17^ This may, in part, explain the slightly better performance in stage 2 compared to stage 1, in which a lower-than-expected proportion of patients with bulbar onset was found. Thus, the relationship between cognition, behavior, and disease stage may be exaggerated by the presence of bulbar symptoms. Levels of depressive symptoms significantly related to behavioral functioning. There may be some overlap between symptoms of depression and behavioral abnormalities, specifically apathy. However, in the present study, higher depression rates were also found in those patients with other behavioral abnormalities, specifically loss of sympathy/empathy and disinhibited behavior. It is possible that depressive symptoms and behavioral features occur concurrently, but further research is required to explore this relationship.

Stage 4 may represent a prognostic disease stage without the same degree of underlying pathology of stages 1 through 3. However, removal of patients in stage 4 without the clinical features of stage 3 results in little change to the outcomes of this study. Certainly, respiratory insufficiency is a key feature of stage 4, and 85% of patients in this stage showed respiratory involvement. Given that the defining characteristics of stage 4 are respiratory insufficiency or feeding intervention because of nutritional deficiency, both of which may have secondary confounding effects on cognition, data were analyzed for stages 1 through 3 separately. The results indicated no significant difference between stages on either cognitive or behavioral measures. The reason may be that stage 4 data are driving the effect, as appears to be most likely in the behavioral data. However, it is important to note that both the Jonckheere-Terpstra and Cochran-Armitage tests are based on the assessment of a monotonic effect. The pattern of results for stages 1 through 3 appears curvilinear; therefore, the analyses lack the necessary power to detect an effect, and the decline from stage 2 to 3 is not sufficient to overcome the removal of stage 4.

Strengths of this study include its prospective multicenter design, a large sample size, and a clinically representative sample. Therefore, the results of this study have good generalizability. However, an important limitation of this study is its cross-sectional design. This restricts the ability to fully explore how cognitive and behavioral symptoms evolve as patients transition to later stages of the disease. To do so, a longitudinal study is required to track patients' cognitive and behavioral performance in line with disease progression. In addition, it is possible that patients with lower cognitive functioning and more severe behavioral abnormalities may have been less likely to participate. Thus, it may be that the present results underestimate the prevalence of neuropsychological impairment across disease stages.

Cognitive and behavioral impairment is common in patients with ALS and present in all stages of the disease. ALS-specific functions (executive, language, and fluency) and behavior are associated with clinical stage as defined by the King's staging system, whereas ALS-nonspecific functions (memory, visuospatial) are not. Measures of cognitive and behavioral change should be included in the diagnostic criteria for ALS and should be incorporated in future staging systems.

## Glossary

ALS: amyotrophic lateral sclerosis
ALSFRS-R: ALS Functional Rating Scale–Revised
ECAS: Edinburgh Cognitive and Behavioural ALS Screen
